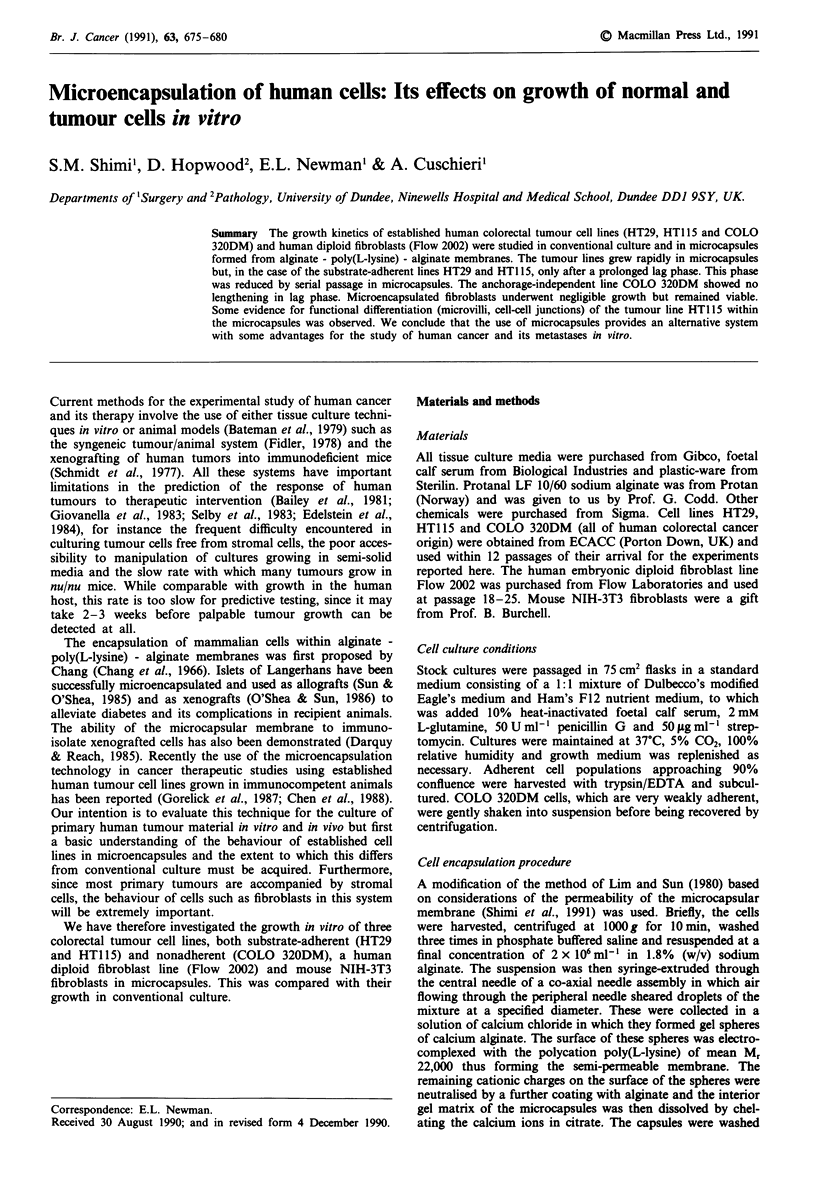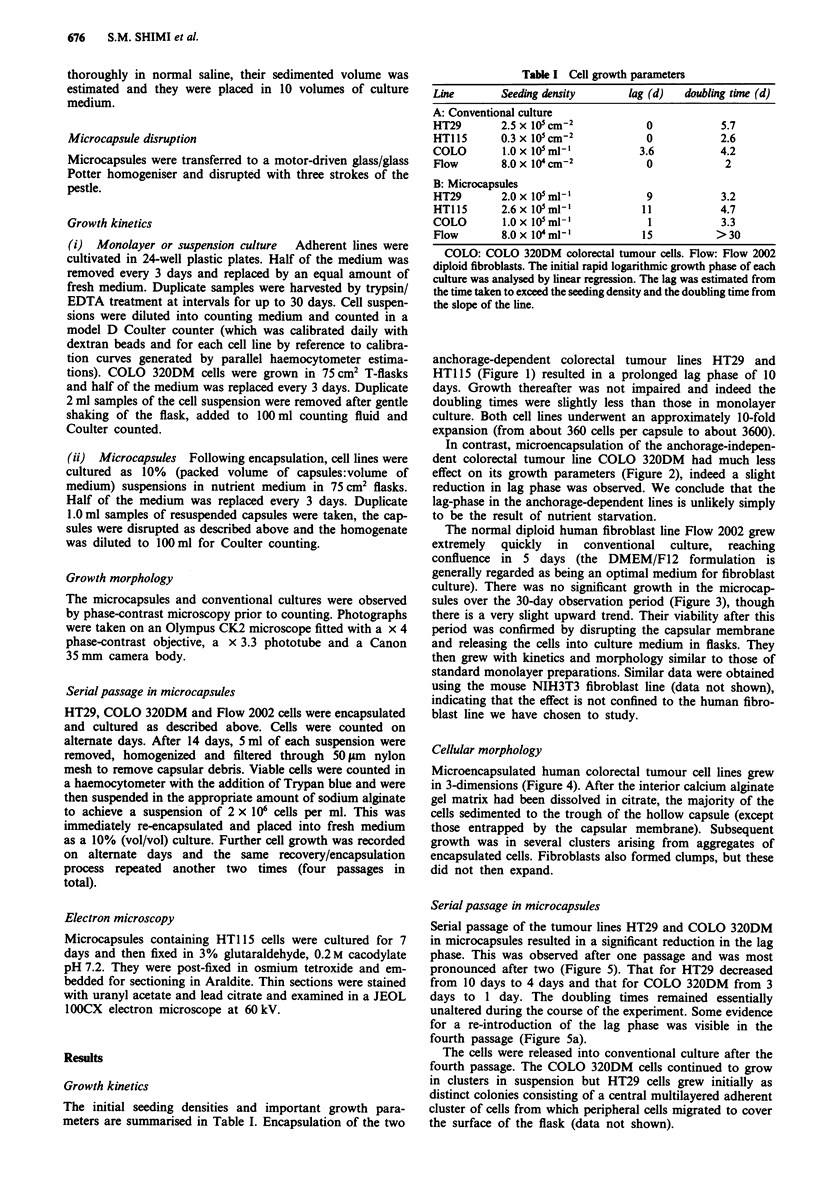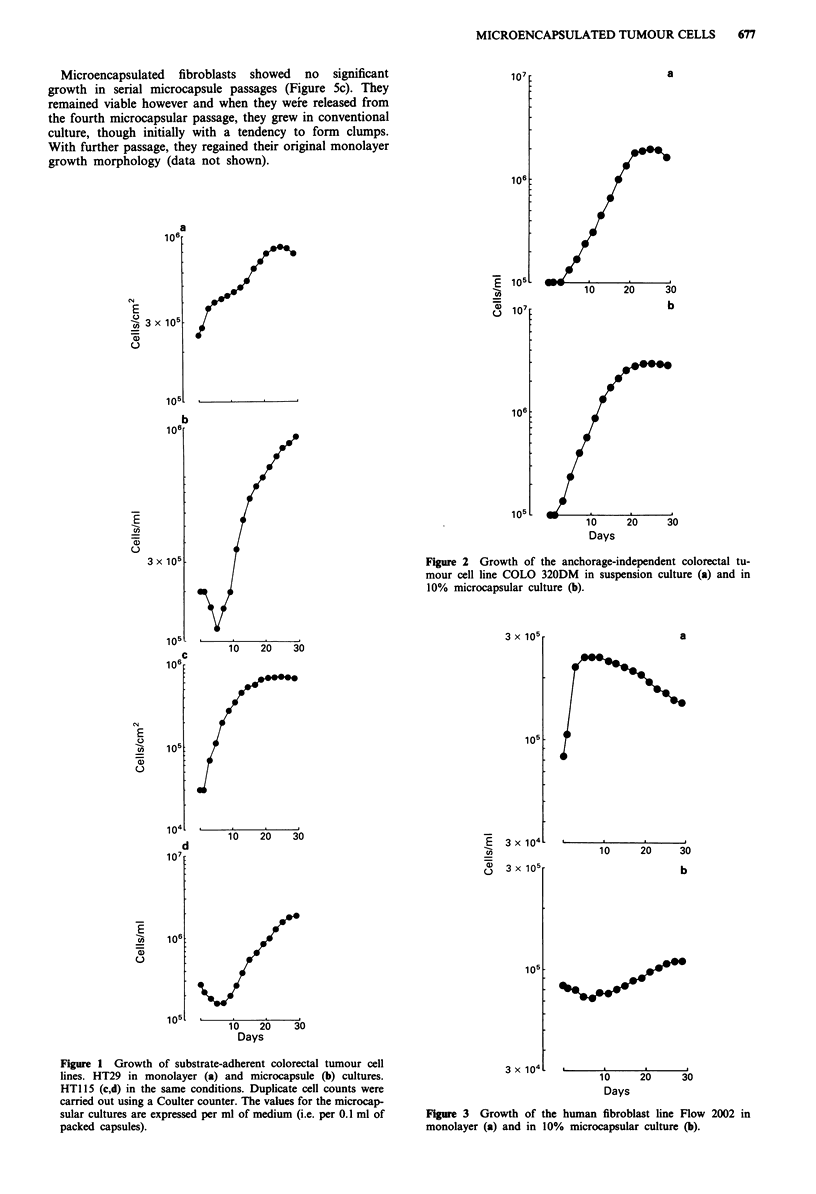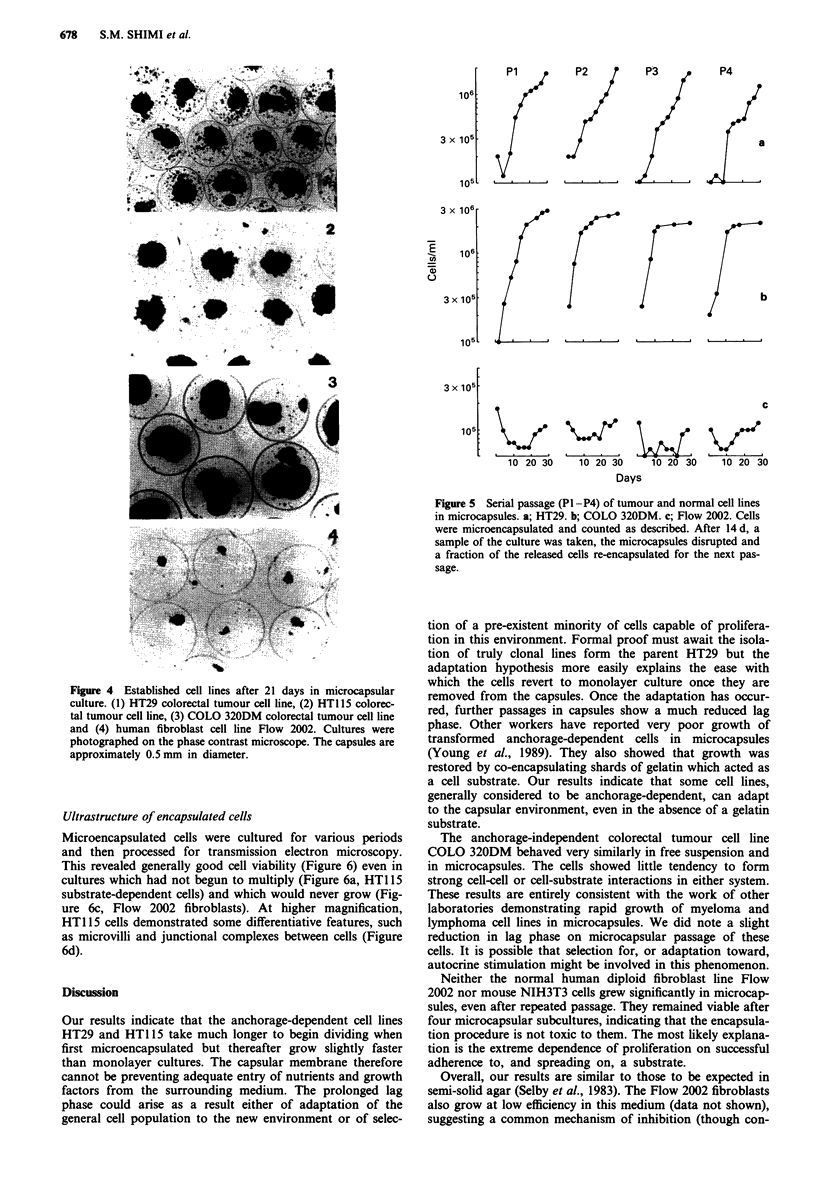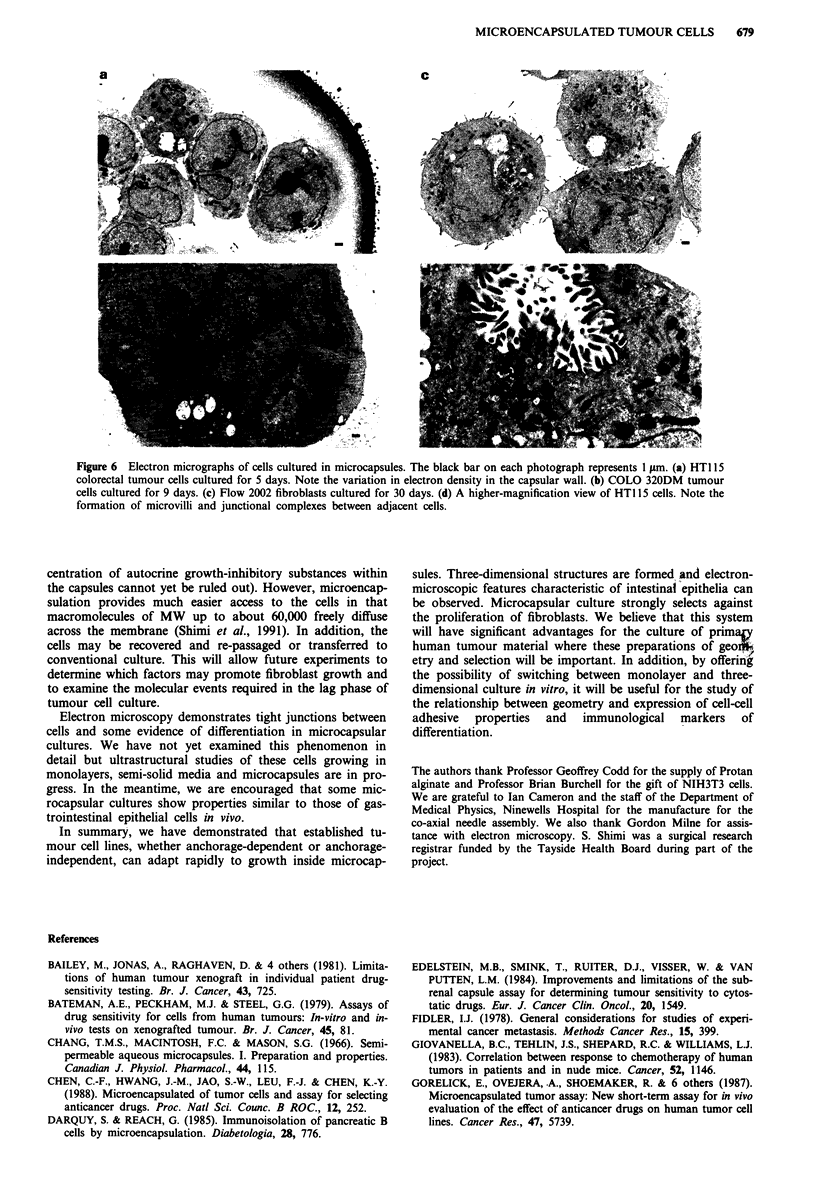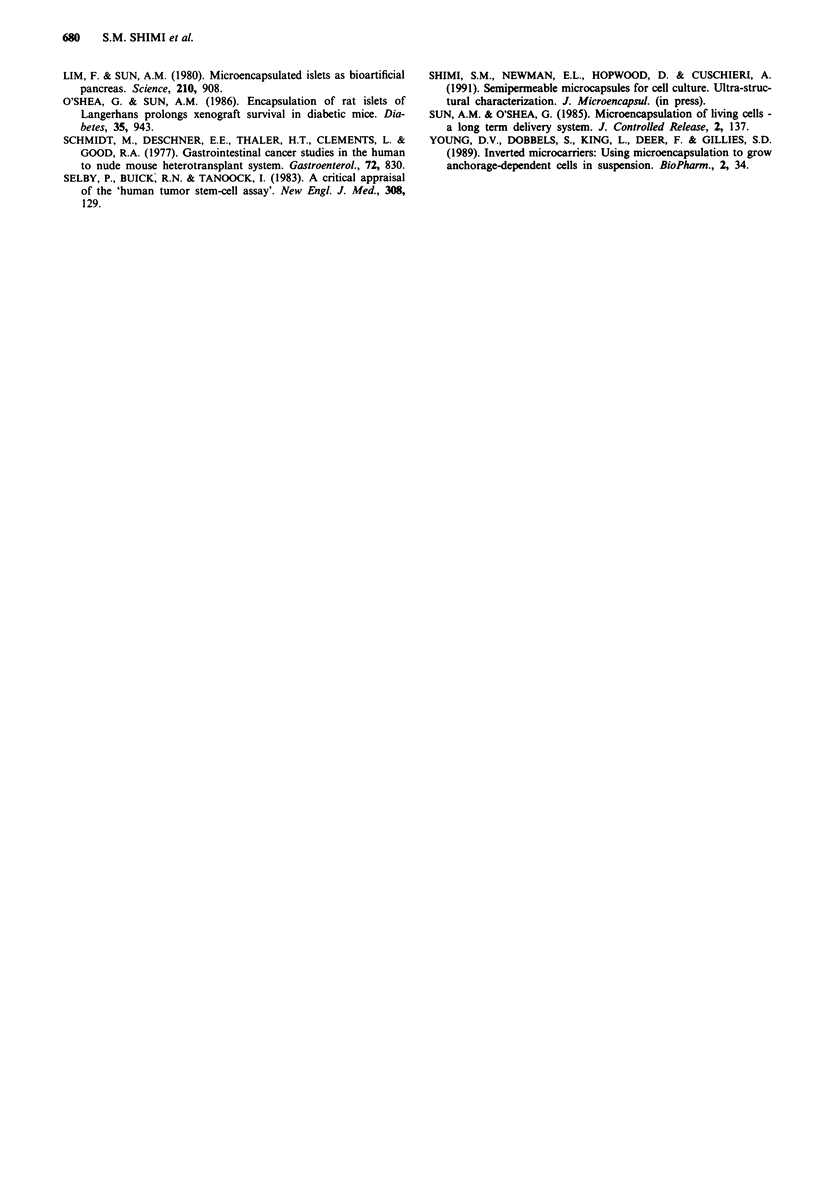# Microencapsulation of human cells: its effects on growth of normal and tumour cells in vitro.

**DOI:** 10.1038/bjc.1991.154

**Published:** 1991-05

**Authors:** S. M. Shimi, D. Hopwood, E. L. Newman, A. Cuschieri

**Affiliations:** Department of Surgery, University of Dundee, Ninewells Hospital and Medical School, UK.

## Abstract

**Images:**


Br. J. Cancer (1991), 63, 675-680 © Macmillan Press Ltd., 1991

### Microencapsulation of human cells: Its effects on growth of normal and tumour cells *in vitro*

S.M. Shimi[1], D. Hopwood[2], E.L. Newman[1] & A. Cuschieri[1]

*Departments of [1]Surgery and [2]Pathology, University of Dundee, Ninewells Hospital and Medical School, Dundee DD1 9SY, UK.*

**Summary** The growth kinetics of established human colorectal tumour cell lines (HT29, HT115 and COLO 320DM) and human diploid fibroblasts (Flow 2002) were studied in conventional culture and in microcapsules formed from alginate - poly(L-lysine) - alginate membranes. The tumour lines grew rapidly in microcapsules but, in the case of the substrate-adherent lines HT29 and HT115, only after a prolonged lag phase. This phase was reduced by serial passage in microcapsules. The anchorage-independent line COLO 320DM showed no lengthening in lag phase. Microencapsulated fibroblasts underwent negligible growth but remained viable. Some evidence for functional differentiation (microvilli, cell-cell junctions) of the tumour line HT115 within the microcapsules was observed. We conclude that the use of microcapsules provides an alternative system with some advantages for the study of human cancer and its metastases *in vitro*.

Current methods for the experimental study of human cancer and its therapy involve the use of either tissue culture techniques *in vitro* or animal models (Bateman *et al.*, 1979) such as the syngeneic tumour/animal system (Fidler, 1978) and the xenografting of human tumors into immunodeficient mice (Schmidt *et al.*, 1977). All these systems have important limitations in the prediction of the response of human tumours to therapeutic intervention (Bailey *et al.*, 1981; Giovanella *et al.*, 1983; Selby *et al.*, 1983; Edelstein *et al.*, 1984), for instance the frequent difficulty encountered in culturing tumour cells free from stromal cells, the poor accessibility to manipulation of cultures growing in semi-solid media and the slow rate with which many tumours grow in *nu*/*nu* mice. While comparable with growth in the human host, this rate is too slow for predictive testing, since it may take 2-3 weeks before palpable tumour growth can be detected at all.

The encapsulation of mammalian cells within alginate - poly(L-lysine) - alginate membranes was first proposed by Chang (Chang *et al.*, 1966). Islets of Langerhans have been successfully microencapsulated and used as allografts (Sun & O'Shea, 1985) and as xenografts (O'Shea & Sun, 1986) to alleviate diabetes and its complications in recipient animals. The ability of the microcapsular membrane to immunoisolate xenografted cells has also been demonstrated (Darquy & Reach, 1985). Recently the use of the microencapsulation technology in cancer therapeutic studies using established human tumour cell lines grown in immunocompetent animals has been reported (Gorelick *et al.*, 1987; Chen *et al.*, 1988). Our intention is to evaluate this technique for the culture of primary human tumour material *in vitro* and *in vivo* but first a basic understanding of the behaviour of established cell lines in microencapsules and the extent to which this differs from conventional culture must be acquired. Furthermore, since most primary tumours are accompanied by stromal cells, the behaviour of cells such as fibroblasts in this system will be extremely important.

We have therefore investigated the growth *in vitro* of three colorectal tumour cell lines, both substrate-adherent (HT29 and HT115) and nonadherent (COLO 320DM), a human diploid fibroblast line (Flow 2002) and mouse NIH-3T3 fibroblasts in microcapsules. This was compared with their growth in conventional culture.

Correspondence: E.L. Newman.
Received 30 August 1990; and in revised form 4 December 1990.

### Materials and methods

### *Materials*

All tissue culture media were purchased from Gibco, foetal calf serum from Biological Industries and plastic-ware from Sterilin. Protanal LF 10/60 sodium alginate was from Protan (Norway) and was given to us by Prof. G. Codd. Other chemicals were purchased from Sigma. Cell lines HT29, HT115 and COLO 320DM (all of human colorectal cancer origin) were obtained from ECACC (Porton Down, UK) and used within 12 passages of their arrival for the experiments reported here. The human embryonic diploid fibroblast line Flow 2002 was purchased from Flow Laboratories and used at passage 18-25. Mouse NIH-3T3 fibroblasts were a gift from Prof. B. Burchell.

### *Cell culture conditions*

Stock cultures were passaged in 75 $cm^2$ flasks in a standard medium consisting of a 1:1 mixture of Dulbecco's modified Eagle's medium and Ham's F12 nutrient medium, to which was added 10% heat-inactivated foetal calf serum, 2 mM L-glutamine, 50 U $ml^{-1}$ penicillin G and 50 $\mu g\ ml^{-1}$ streptomycin. Cultures were maintained at 37°C, 5% $CO_2$, 100% relative humidity and growth medium was replenished as necessary. Adherent cell populations approaching 90% confluence were harvested with trypsin/EDTA and subcultured. COLO 320DM cells, which are very weakly adherent, were gently shaken into suspension before being recovered by centrifugation.

### *Cell encapsulation procedure*

A modification of the method of Lim and Sun (1980) based on considerations of the permeability of the microcapsular membrane (Shimi *et al.*, 1991) was used. Briefly, the cells were harvested, centrifuged at 1000 *g* for 10 min, washed three times in phosphate buffered saline and resuspended at a final concentration of $2 \times 10^6\ ml^{-1}$ in 1.8% (w/v) sodium alginate. The suspension was then syringe-extruded through the central needle of a co-axial needle assembly in which air flowing through the peripheral needle sheared droplets of the mixture at a specified diameter. These were collected in a solution of calcium chloride in which they formed gel spheres of calcium alginate. The surface of these spheres was electrocomplexed with the polycation poly(L-lysine) of mean $M_r$ 22,000 thus forming the semi-permeable membrane. The remaining cationic charges on the surface of the spheres were neutralised by a further coating with alginate and the interior gel matrix of the microcapsules was then dissolved by chelating the calcium ions in citrate. The capsules were washed thoroughly in normal saline, their sedimented volume was estimated and they were placed in 10 volumes of culture medium.

*Microcapsule disruption*

Microcapsules were transferred to a motor-driven glass/glass Potter homogeniser and disrupted with three strokes of the pestle.

*Growth kinetics*

*(i) Monolayer or suspension culture* Adherent lines were cultivated in 24-well plastic plates. Half of the medium was removed every 3 days and replaced by an equal amount of fresh medium. Duplicate samples were harvested by trypsin/EDTA treatment at intervals for up to 30 days. Cell suspensions were diluted into counting medium and counted in a model D Coulter counter (which was calibrated daily with dextran beads and for each cell line by reference to calibration curves generated by parallel haemocytometer estimations). COLO 320DM cells were grown in 75 $cm^2$ T-flasks and half of the medium was replaced every 3 days. Duplicate 2 ml samples of the cell suspension were removed after gentle shaking of the flask, added to 100 ml counting fluid and Coulter counted.

*(ii) Microcapsules* Following encapsulation, cell lines were cultured as 10% (packed volume of capsules:volume of medium) suspensions in nutrient medium in 75 $cm^2$ flasks. Half of the medium was replaced every 3 days. Duplicate 1.0 ml samples of resuspended capsules were taken, the capsules were disrupted as described above and the homogenate was diluted to 100 ml for Coulter counting.

*Growth morphology*

The microcapsules and conventional cultures were observed by phase-contrast microscopy prior to counting. Photographs were taken on an Olympus CK2 microscope fitted with a $\times 4$ phase-contrast objective, a $\times 3.3$ phototube and a Canon 35 mm camera body.

*Serial passage in microcapsules*

HT29, COLO 320DM and Flow 2002 cells were encapsulated and cultured as described above. Cells were counted on alternate days. After 14 days, 5 ml of each suspension were removed, homogenized and filtered through 50 $\mu m$ nylon mesh to remove capsular debris. Viable cells were counted in a haemocytometer with the addition of Trypan blue and were then suspended in the appropriate amount of sodium alginate to achieve a suspension of $2 \times 10^6$ cells per ml. This was immediately re-encapsulated and placed into fresh medium as a 10% (vol/vol) culture. Further cell growth was recorded on alternate days and the same recovery/encapsulation process repeated another two times (four passages in total).

*Electron microscopy*

Microcapsules containing HT115 cells were cultured for 7 days and then fixed in 3% glutaraldehyde, 0.2 M cacodylate pH 7.2. They were post-fixed in osmium tetroxide and embedded for sectioning in Araldite. Thin sections were stained with uranyl acetate and lead citrate and examined in a JEOL 100CX electron microscope at 60 kV.

### Results

*Growth kinetics*

The initial seeding densities and important growth parameters are summarised in Table I. Encapsulation of the two anchorage-dependent colorectal tumour lines HT29 and HT115 (Figure 1) resulted in a prolonged lag phase of 10 days. Growth thereafter was not impaired and indeed the doubling times were slightly less than those in monolayer culture. Both cell lines underwent an approximately 10-fold expansion (from about 360 cells per capsule to about 3600).

**Table I** Cell growth parameters

| *Line* | *Seeding density* | *lag (d)* | *doubling time (d)* |
|---|---|---|---|
| A: Conventional culture | | | |
| HT29 | $2.5 \times 10^5 cm^{-2}$ | 0 | 5.7 |
| HT115 | $0.3 \times 10^5 cm^{-2}$ | 0 | 2.6 |
| COLO | $1.0 \times 10^5 ml^{-1}$ | 3.6 | 4.2 |
| Flow | $8.0 \times 10^4 cm^{-2}$ | 0 | 2 |
| B: Microcapsules | | | |
| HT29 | $2.0 \times 10^5 ml^{-1}$ | 9 | 3.2 |
| HT115 | $2.6 \times 10^5 ml^{-1}$ | 11 | 4.7 |
| COLO | $1.0 \times 10^5 ml^{-1}$ | 1 | 3.3 |
| Flow | $8.0 \times 10^4 ml^{-1}$ | 15 | >30 |

COLO: COLO 320DM colorectal tumour cells. Flow: Flow 2002 diploid fibroblasts. The initial rapid logarithmic growth phase of each culture was analysed by linear regression. The lag was estimated from the time taken to exceed the seeding density and the doubling time from the slope of the line.

In contrast, microencapsulation of the anchorage-independent colorectal tumour line COLO 320DM had much less effect on its growth parameters (Figure 2), indeed a slight reduction in lag phase was observed. We conclude that the lag-phase in the anchorage-dependent lines is unlikely simply to be the result of nutrient starvation.

The normal diploid human fibroblast line Flow 2002 grew extremely quickly in conventional culture, reaching confluence in 5 days (the DMEM/F12 formulation is generally regarded as being an optimal medium for fibroblast culture). There was no significant growth in the microcapsules over the 30-day observation period (Figure 3), though there is a very slight upward trend. Their viability after this period was confirmed by disrupting the capsular membrane and releasing the cells into culture medium in flasks. They then grew with kinetics and morphology similar to those of standard monolayer preparations. Similar data were obtained using the mouse NIH3T3 fibroblast line (data not shown), indicating that the effect is not confined to the human fibroblast line we have chosen to study.

*Cellular morphology*

Microencapsulated human colorectal tumour cell lines grew in 3-dimensions (Figure 4). After the interior calcium alginate gel matrix had been dissolved in citrate, the majority of the cells sedimented to the trough of the hollow capsule (except those entrapped by the capsular membrane). Subsequent growth was in several clusters arising from aggregates of encapsulated cells. Fibroblasts also formed clumps, but these did not then expand.

*Serial passage in microcapsules*

Serial passage of the tumour lines HT29 and COLO 320DM in microcapsules resulted in a significant reduction in the lag phase. This was observed after one passage and was most pronounced after two (Figure 5). That for HT29 decreased from 10 days to 4 days and that for COLO 320DM from 3 days to 1 day. The doubling times remained essentially unaltered during the course of the experiment. Some evidence for a re-introduction of the lag phase was visible in the fourth passage (Figure 5a).

The cells were released into conventional culture after the fourth passage. The COLO 320DM cells continued to grow in clusters in suspension but HT29 cells grew initially as distinct colonies consisting of a central multilayered adherent cluster of cells from which peripheral cells migrated to cover the surface of the flask (data not shown).

Microencapsulated fibroblasts showed no significant growth in serial microcapsule passages (Figure 5c). They remained viable however and when they were released from the fourth microcapsular passage, they grew in conventional culture, though initially with a tendency to form clumps. With further passage, they regained their original monolayer growth morphology (data not shown).

**Figure 1** Growth of substrate-adherent colorectal tumour cell lines. HT29 in monolayer (**a**) and microcapsule (**b**) cultures. HT115 (**c**,**d**) in the same conditions. Duplicate cell counts were carried out using a Coulter counter. The values for the microcapsular cultures are expressed per ml of medium (i.e. per 0.1 ml of packed capsules).

**Figure 2** Growth of the anchorage-independent colorectal tumour cell line COLO 320DM in suspension culture (**a**) and in 10% microcapsular culture (**b**).

**Figure 3** Growth of the human fibroblast line Flow 2002 in monolayer (**a**) and in 10% microcapsular culture (**b**).

**Figure 4** Established cell lines after 21 days in microcapsular culture. (1) HT29 colorectal tumour cell line, (2) HT115 colorectal tumour cell line, (3) COLO 320DM colorectal tumour cell line and (4) human fibroblast cell line Flow 2002. Cultures were photographed on the phase contrast microscope. The capsules are approximately 0.5 mm in diameter.

**Figure 5** Serial passage (P1–P4) of tumour and normal cell lines in microcapsules. **a**; HT29. **b**; COLO 320DM. **c**; Flow 2002. Cells were microencapsulated and counted as described. After 14 d, a sample of the culture was taken, the microcapsules disrupted and a fraction of the released cells re-encapsulated for the next passage.

*Ultrastructure of encapsulated cells*

Microencapsulated cells were cultured for various periods and then processed for transmission electron microscopy. This revealed generally good cell viability (Figure 6) even in cultures which had not begun to multiply (Figure 6a, HT115 substrate-dependent cells) and which would never grow (Figure 6c, Flow 2002 fibroblasts). At higher magnification, HT115 cells demonstrated some differentiative features, such as microvilli and junctional complexes between cells (Figure 6d).

### Discussion

Our results indicate that the anchorage-dependent cell lines HT29 and HT115 take much longer to begin dividing when first microencapsulated but thereafter grow slightly faster than monolayer cultures. The capsular membrane therefore cannot be preventing adequate entry of nutrients and growth factors from the surrounding medium. The prolonged lag phase could arise as a result either of adaptation of the general cell population to the new environment or of selection of a pre-existent minority of cells capable of proliferation in this environment. Formal proof must await the isolation of truly clonal lines form the parent HT29 but the adaptation hypothesis more easily explains the ease with which the cells revert to monolayer culture once they are removed from the capsules. Once the adaptation has occurred, further passages in capsules show a much reduced lag phase. Other workers have reported very poor growth of transformed anchorage-dependent cells in microcapsules (Young *et al.*, 1989). They also showed that growth was restored by co-encapsulating shards of gelatin which acted as a cell substrate. Our results indicate that some cell lines, generally considered to be anchorage-dependent, can adapt to the capsular environment, even in the absence of a gelatin substrate.

The anchorage-independent colorectal tumour cell line COLO 320DM behaved very similarly in free suspension and in microcapsules. The cells showed little tendency to form strong cell-cell or cell-substrate interactions in either system. These results are entirely consistent with the work of other laboratories demonstrating rapid growth of myeloma and lymphoma cell lines in microcapsules. We did note a slight reduction in lag phase on microcapsular passage of these cells. It is possible that selection for, or adaptation toward, autocrine stimulation might be involved in this phenomenon.

Neither the normal human diploid fibroblast line Flow 2002 nor mouse NIH3T3 cells grew significantly in microcapsules, even after repeated passage. They remained viable after four microcapsular subcultures, indicating that the encapsulation procedure is not toxic to them. The most likely explanation is the extreme dependence of proliferation on successful adherence to, and spreading on, a substrate.

Overall, our results are similar to those to be expected in semi-solid agar (Selby *et al.*, 1983). The Flow 2002 fibroblasts also grow at low efficiency in this medium (data not shown), suggesting a common mechanism of inhibition (though con-

**Figure 6** Electron micrographs of cells cultured in microcapsules. The black bar on each photograph represents 1 μm. (**a**) HT115 colorectal tumour cells cultured for 5 days. Note the variation in electron density in the capsular wall. (**b**) COLO 320DM tumour cells cultured for 9 days. (**c**) Flow 2002 fibroblasts cultured for 30 days. (**d**) A higher-magnification view of HT115 cells. Note the formation of microvilli and junctional complexes between adjacent cells.

centration of autocrine growth-inhibitory substances within the capsules cannot yet be ruled out). However, microencapsulation provides much easier access to the cells in that macromolecules of MW up to about 60,000 freely diffuse across the membrane (Shimi *et al.*, 1991). In addition, the cells may be recovered and re-passaged or transferred to conventional culture. This will allow future experiments to determine which factors may promote fibroblast growth and to examine the molecular events required in the lag phase of tumour cell culture.

Electron microscopy demonstrates tight junctions between cells and some evidence of differentiation in microcapsular cultures. We have not yet examined this phenomenon in detail but ultrastructural studies of these cells growing in monolayers, semi-solid media and microcapsules are in progress. In the meantime, we are encouraged that some microcapsular cultures show properties similar to those of gastrointestinal epithelial cells *in vivo*.

In summary, we have demonstrated that established tumour cell lines, whether anchorage-dependent or anchorage-independent, can adapt rapidly to growth inside microcapsules. Three-dimensional structures are formed and electron-microscopic features characteristic of intestinal epithelia can be observed. Microcapsular culture strongly selects against the proliferation of fibroblasts. We believe that this system will have significant advantages for the culture of primary human tumour material where these preparations of geometry and selection will be important. In addition, by offering the possibility of switching between monolayer and three-dimensional culture *in vitro*, it will be useful for the study of the relationship between geometry and expression of cell-cell adhesive properties and immunological markers of differentiation.

The authors thank Professor Geoffrey Codd for the supply of Protan alginate and Professor Brian Burchell for the gift of NIH3T3 cells. We are grateful to Ian Cameron and the staff of the Department of Medical Physics, Ninewells Hospital for the manufacture for the co-axial needle assembly. We also thank Gordon Milne for assistance with electron microscopy. S. Shimi was a surgical research registrar funded by the Tayside Health Board during part of the project.

### References

BAILEY, M., JONAS, A., RAGHAVEN, D. & 4 others (1981). Limitations of human tumour xenograft in individual patient drug-sensitivity testing. *Br. J. Cancer*, **43**, 725.

BATEMAN, A.E., PECKHAM, M.J. & STEEL, G.G. (1979). Assays of drug sensitivity for cells from human tumours: *In-vitro* and *in-vivo* tests on xenografted tumour. *Br. J. Cancer*, **45**, 81.

CHANG, T.M.S., MACINTOSH, F.C. & MASON, S.G. (1966). Semi-permeable aqueous microcapsules. I. Preparation and properties. *Canadian J. Physiol. Pharmacol.*, **44**, 115.

CHEN, C.-F., HWANG, J.-M., JAO, S.-W., LEU, F.-J. & CHEN, K.-Y. (1988). Microencapsulated of tumor cells and assay for selecting anticancer drugs. *Proc. Natl Sci. Counc. B ROC.*, **12**, 252.

DARQUY, S. & REACH, G. (1985). Immunoisolation of pancreatic B cells by microencapsulation. *Diabetologia*, **28**, 776.

EDELSTEIN, M.B., SMINK, T., RUITER, D.J., VISSER, W. & VAN PUTTEN, L.M. (1984). Improvements and limitations of the subrenal capsule assay for determining tumour sensitivity to cytostatic drugs. *Eur. J. Cancer Clin. Oncol.*, **20**, 1549.

FIDLER, I.J. (1978). General considerations for studies of experimental cancer metastasis. *Methods Cancer Res.*, **15**, 399.

GIOVANELLA, B.C., TEHLIN, J.S., SHEPARD, R.C. & WILLIAMS, L.J. (1983). Correlation between response to chemotherapy of human tumors in patients and in nude mice. *Cancer*, **52**, 1146.

GORELICK, E., OVEJERA, A., SHOEMAKER, R. & 6 others (1987). Microencapsulated tumor assay: New short-term assay for *in vivo* evaluation of the effect of anticancer drugs on human tumor cell lines. *Cancer Res.*, **47**, 5739.

LIM, F. & SUN, A.M. (1980). Microencapsulated islets as bioartificial pancreas. *Science*, **210**, 908.

O'SHEA, G. & SUN, A.M. (1986). Encapsulation of rat islets of Langerhans prolongs xenograft survival in diabetic mice. *Diabetes*, **35**, 943.

SCHMIDT, M., DESCHNER, E.E., THALER, H.T., CLEMENTS, L. & GOOD, R.A. (1977). Gastrointestinal cancer studies in the human to nude mouse heterotransplant system. *Gastroenterol.*, **72**, 830.

SELBY, P., BUICK, R.N. & TANOOCK, I. (1983). A critical appraisal of the 'human tumor stem-cell assay'. *New Engl. J. Med.*, **308**, 129.

SHIMI, S.M., NEWMAN, E.L., HOPWOOD, D. & CUSCHIERI, A. (1991). Semipermeable microcapsules for cell culture. Ultra-structural characterization. *J. Microencapsul.* (in press).

SUN, A.M. & O'SHEA, G. (1985). Microencapsulation of living cells - a long term delivery system. *J. Controlled Release*, **2**, 137.

YOUNG, D.V., DOBBELS, S., KING, L., DEER, F. & GILLIES, S.D. (1989). Inverted microcarriers: Using microencapsulation to grow anchorage-dependent cells in suspension. *BioPharm.*, **2**, 34.